# Influencing factors on serum 25-hydroxyvitamin D_3_ levels in Japanese chronic hepatitis C patients

**DOI:** 10.1186/s12879-015-1020-y

**Published:** 2015-08-19

**Authors:** Masanori Atsukawa, Akihito Tsubota, Noritomo Shimada, Kai Yoshizawa, Hiroshi Abe, Toru Asano, Yusuke Ohkubo, Masahiro Araki, Tadashi Ikegami, Chisa Kondo, Norio Itokawa, Ai Nakagawa, Taeang Arai, Yoko Matsushita, Katsuhisa Nakatsuka, Tomomi Furihata, Yoshimichi Chuganji, Yasushi Matsuzaki, Yoshio Aizawa, Katsuhiko Iwakiri

**Affiliations:** Nippon Medical School Chiba Hokusoh Hospital, 1715, Inzai, Chiba Japan; Core Research Facilities for Basic Science, Research Center for Medical Sciences, Jikei University School of Medicine, 3-25-8, Minato-ku, Tokyo Japan; Chiba Tokushukai Hospital, 2-11-1 Takanedai, Funabashi, Chiba Japan; Machida Municipal Hospital, 2-15-41 Asahi-cho, Machida, Tokyo Japan; Jikei University School of Medicine Katsusika Medical Center, 6-41-2 Aoto, Katsushika, Tokyo Japan; Tokyo Metropolitan Bokutoh Hospital, 4-23-15 Koutoubashi, Sumida, Tokyo Japan; Saiseikai Yokohamashi Tobu Hospital, 3-6-1 shimosueyoshi, Tsurumi, Kanagawa Japan; Ibaraki Central Hospital, Kasama, 6528, Koihuchi, Ibaraki Japan; Tokyo Medical University, Ibaraki Medical Center, 3-20-1 amichochuo, Inashiki, Ibaraki Japan; Nippon Medical School, 1-1-5, Bunkyo, Tokyo Japan; Laboratory of Pharmacology and Toxicology, Graduate School of Pharmaceutical Sciences, Chiba University, 1-8-1 Inohana, Chuo-ku, Chiba Japan

**Keywords:** Chronic hepatitis C, Vitamin D, 25-hydroxyvitamin D_3_

## Abstract

**Background:**

Serum 25-hydroxyvitamin D_3_ levels are generally lower in chronic hepatitis C patients than in healthy individuals. The purpose of this study is to clarify the factors which affect serum 25-hydroxyvitamin D_3_ levels using data obtained from Japanese chronic hepatitis C patients.

**Methods:**

The subjects were 619 chronic hepatitis C patients. Serum 25-hydroxyvitamin D_3_ levels were measured by using double-antibody radioimmunoassay between April 2009 and August 2014. Serum 25-hydroxyvitamin D_3_ levels of 20 ng/mL or less were classified as vitamin D deficiency, and those with serum 25-hydroxyvitamin D_3_ levels of 30 ng/mL or more as vitamin D sufficiency. The relationship between patient-related factors and serum 25-hydroxyvitamin D_3_ levels was analyzed.

**Results:**

The cohort consisted of 305 females and 314 males, aged between 18 and 89 years (median, 63 years). The median serum 25-hydroxyvitamin D_3_ level was 21 ng/mL (range, 6–61 ng/mL). On the other hand, the median serum 25-hydroxyvitamin D_3_ level in the healthy subjects was 25 ng/mL (range, 7–52), being significantly higher than that those in 80 chronic hepatitis C patients matched for age, gender, and season (*p* = 1.16 × 10^−8^). In multivariate analysis, independent contributors to serum 25-hydroxyvitamin D_3_ deficiency were as follows: female gender (*p* = 2.03 × 10^−4^, odds ratio = 2.290, 95 % confidence interval = 1.479–3.545), older age (*p* = 4.30 × 10^−4^, odds ratio = 1.038, 95 % confidence interval = 1.017–1.060), cold season (*p* = 0.015, odds ratio = 1.586, 95 % confidence interval = 1.095–2.297), and low hemoglobin level (*p* = 0.037, odds ratio = 1.165, 95 % confidence interval = 1.009–1.345). By contrast, independent contributors to serum 25-hydroxyvitamin D_3_ sufficiency were male gender (*p* = 0.001, odds ratio = 3.400, 95 % confidence interval = 1.635–7.069), warm season (*p* = 0.014, odds ratio = 1.765, 95 % confidence interval = 1.117–2.789) and serum albumin (*p* = 0.016, OR = 2.247, 95 % CI = 1.163–4.342).

**Conclusions:**

Serum 25-hydroxyvitamin D_3_ levels in chronic hepatitis C Japanese patients were influenced by gender, age, hemoglobin level, albumin and the season of measurement.

## Background

Chronic hepatitis C (CHC) affects 170 million people worldwide [1]. Liver cirrhosis and hepatocellular carcinoma (HCC) are known to develop if CHC is left untreated; therefore, hepatitis C virus (HCV) needs to be eliminated from CHC patients [2]. In recent years, remarkable progress has been made in the treatment of CHC. The sustained virologic response (SVR) rate in patients with genotype 1 CHC, which is difficult to treat, has been reported to improve to approximately 80 % by pegylated interferon (IFN)/ribavirin combined with protease inhibitors [3–5]. Furthermore, direct-acting antiviral agents such as NS3/4A protease inhibitors, NS5B polymerase inhibitors, and NS5A inhibitors have a strong therapeutic effect by directly inhibiting the proliferation of HCV, and thus is expected to be the mainstream treatment for CHC in the future [6–9].

The classical active form of vitamin D plays a major role in increasing the serum calcium concentration by promoting absorption of calcium from the intestinal tract or increasing bone resorption [10]. In recent years, reports have been accumulating on the involvement of serum vitamin D level in various diseases. The mortality from cardiovascular disease is low in patients with high serum vitamin D levels [11]. Also, low serum vitamin levels have been related to the incidence of various cancers, including colon cancer [12], breast cancer [13], and prostate cancer [14, 15]. Low serum vitamin D levels are suggested to aggravate insulin resistance [16], which can be improved with vitamin D supplements [17]. Low serum vitamin D levels are also observed in patients who have undergone gastrectomy, suggesting that the reduced level is causally related to insufficient vitamin D absorption or intake [18, 19]. Serum vitamin D levels have been also reported to be closely correlated with CHC.

Vitamin D is synthesized mainly in the skin as well as being ingested from food. This synthesis of vitamin D in the skin declines with age. With ultraviolet irradiation, 7-dehydrocholesterol in the skin is converted to pre-vitamin D_3_. Position 25 is hydroxylated in the liver and metabolized to 25-hydroxyvitamin D_3_. Position 1α is hydroxylated in the kidneys to form active 1,25-dihydroxyvitamin D_3_, which binds to vitamin D receptors. In a related pathway, vitamin D is converted into various metabolites, which bind to vitamin D-binding protein and circulate stably in blood. Among the circulating metabolites, 25-hydroxyvitamin D_3_ is the most stable and has a long half-life in blood. Therefore, the serum 25-hydroxyvitamin D_3_ level is often used as a representative value for the serum vitamin D level. Serum 25-hydroxyvitamin D_3_ levels are lower in CHC patients than in healthy individuals, and decrease with the progression of liver fibrosis [20, 21]. In IFN-based therapy for CHC, serum vitamin D levels influence the therapeutic efficacy [20, 22–24].

Serum 25-hydroxyvitamin D_3_ levels are thought to differ by country and region. For instance, they tend to be lower at high latitudes. A study of healthy people in Norway showed that the mean serum 25-hydroxyvitamin D_3_ level was approximately 20 ng/ml, and that the percentage of subjects with sufficient serum 25-hydroxyvitamin D_3_ levels was only 9 % [25]. However, the deficiency of serum 25-hydroxyvitamin D_3_ level cannot be explained by the difference in latitudes alone. In fact, the serum 25-hydroxyvitamin D_3_ level was reported to be lower than 30 ng/mL in more than 50 % of young people in Hawaii despite sufficient exposure to sunlight [26]. The discrepancy may be attributable to racial difference and dietary lifestyle.

There have been few studies concerning factors correlated with serum 25-hydroxyvitamin D_3_ levels for CHC patients in East Asia, where there are four distinct seasons in temperate latitudes. The purpose of this study was to investigate serum 25-hydroxyvitamin D_3_ levels and to analyze factors influencing these levels using data obtained Japanese chronic hepatitis C patients.

## Methods

### Subjects

The subjects were CHC patients who visited the Nippon Medical School Chiba Hokusoh Hospital, Nippon Medical School, Tokushukai Chiba Hospital, Jikei University School of Medicine Katsusika Medical Center, Jikei University School of Medicine Kashiwa Hospital, Ibaraki Central Hospital, Tokyo Metropolitan Bokutoh Hospital, Tokyo Medical University Ibaraki Medical Center, Machida Municipal Hospital, and Saiseikai Yokohamashi Tobu Hospital between April 2009 and August 2014. All were positive for HCV RNA based on real-time polymerase chain reaction (PCR)-based assay. The analysis was exclusively done on candidates for antiviral treatment, and thus was limited to those infected with HCV genotype 1, who are difficult to treat and account for a large portion of CHC patients in Japan. To reduce the influence of patient state on serum vitamin D levels, only patients with a score of 0 or 1 based on the Eastern Cooperative Oncology Group Performance Status criteria were selected as subjects. Exclusion criteria were hepatitis B surface antigen positivity, human immunodeficiency virus antigen positivity, the presence of other chronic liver diseases such as autoimmune hepatitis or primary biliary cirrhosis, decompensated cirrhosis, taking drugs and/or supplements containing vitamin D, and being under anti-cancer treatment. Eighty healthy volunteers recruited from April 2009 and August 2014, matched for gender, age and season, were enrolled as controls. The study protocol followed the ethical guidelines established in accordance with the 2008 Declaration of Helsinki and was approved by the Ethics Committee of Nippon Medical School Chiba Hokusoh Hospital. All patients provided written informed consent.

### Laboratory methods

Hematological and biochemical tests were performed, and clotting factors and tumor markers were measured for all patients. Serum 25-hydroxyvitamin D_3_ levels were measured by a double-antibody radioimmunoassay kit (SRL Inc., Tokyo, Japan). Serum 25-hydroxyvitamin D_3_ levels of 20 ng/mL or less were classified as vitamin D deficiency, those of 21–29 ng/mL as vitamin D insufficiency, and those with serum 25-hydroxyvitamin D_3_ levels of 30 ng/mL or more as vitamin D sufficiency [27]. Cases for which serum 25-hydroxyvitamin D_3_ levels were measured between May and October, a period when there are long hours of daylight, were defined as the “warm season” group, and those between November and April, a period when there are short hours of daylight, were defined as the “cold season” group. HCV RNA levels were measured by the real-time PCR method (COBAS® AmpliPrep; Roche Diagnostics, Tokyo). Gene mutations in the core amino acids 70 and 91, and NS5A regions (interferon sensitivity determining region; ISDR) of the HCV genome were determined using the direct sequencing method. Core amino acid 70 was defined as wild type (arginine) or mutant type (glutamine or histidine), and core amino acid 91 was defined as the wild type (leucine) or the mutant type (methionine). Amino acid mutations in the ISDR were defined as wild type (0, 1) or mutant type (2 or more). Genomic DNA was extracted from whole blood using a DNA isolation kit on a MagNA Pure LC Instrument (Roche Diagnostics, Basel, Switzerland). The single nucleotide polymorphism (SNP) rs8099917 near the *interleukin (IL) 28B* gene was determined by real-time detection PCR using the TaqMan® SNP genotyping assay (Applied Biosystems, Foster City, CA). The rs8099917 genotype was classified into two categories: TT and non-TT (TG or GG). The Fib-4 index was calculated as an indicator of fibrosis [28]. A Fib-4 index of greater than 3.25 was defined as advanced liver fibrosis.

### Statistical analyses

Fisher’s exact test and Mann–Whitney *U*-test were performed to compare serum 25-hydroxyvitamin D_3_ levels between the two groups. Logistic regression analysis for univariate comparison was performed to investigate whether each factor influenced the serum 25-hydroxyvitamin D_3_ level. Multiple logistic regression analysis was performed to identify significant, independent factors that influenced serum 25-hydroxyvitamin D_3_ levels. All statistical analyses were performed using IBM SPSS version 17.0 (IBM Japan, Tokyo). The level of statistical significance was set at *p* < 0.05.

## Results

### Background

**Fig. 1:**
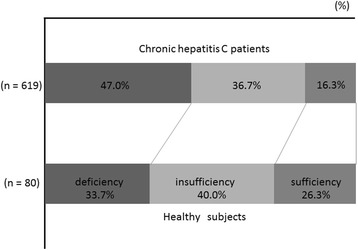
Distribution of serum 25-hydroxyvitamin D_3_ level in chronic hepatitis C patients and healthy subjects. Serum 25-hydroxyvitamin D_3_ levels of 20 ng/mL or less were defined as vitamin D deficiency, those of 21–29 ng/mL were defined as vitamin D insufficiency, and those with serum 25-hydroxyvitamin D_3_ levels of 30 ng/mL or more were defined as vitamin D sufficiency

The 619 subjects consisted of 305 females (49.7 %) and 314 males (50.7 %), aged between 18 and 89 years (median, 63 years; Table 1). The median serum 25-hydroxyvitamin D_3_ level was 21 ng/mL (range, 6–61 ng/mL). Serum 25-hydroxyvitamin D_3_ levels in 257 patients (41.5 %) were measured during the warm season. The median Fib-4 index, an indicator of fibrosis degree in the liver, was 3.12 (range, 0.45–15.97). The number of patients with a Fib-4 index of greater than 3.25, indicative of advanced liver fibrosis [28], was 282 (45.6 %). The percentages of subjects with vitamin D deficiency, insufficiency, and sufficiency were 47.0 % (291 of 619 patients), 36.7 % (227 of 619 patients), and 16.3 % (101 of 619 patients), respectively (Fig. 1). The *IL28B* SNP genotype rs8099917, the most important determinant of the responsiveness to interferon-based therapy, was TT in 356 patients (57.5 %). On the other hand, the median serum 25-hydroxyvitamin D_3_ level in the healthy subjects was 25 ng/mL (range, 7–52), being significantly higher than that those in 80 CHC patients matched for age, gender, and season (*p* = 1.16 × 10^−8^). The percentages of healthy subjects with vitamin D deficiency, insufficiency, and sufficiency were 33.7 % (27 of 80 patients), 40.0 % (32 of 80 patients), and 26.3 % (21 of 80 patients), respectively (Fig. 1).

**Table 1:** Baseline characteristics of 619 patients with chronic hepatitis C

Factors	*n* = 619
Gender (M/F)	314/305
Age (y.o)	63 (18–89)
Body weight (kg)	59.8 (33.0–115.8)
BMI (kg/m^2^)	23.86 (15.48–37.81)
Fib-4 Index	3.12 (0.45–15.97)
Season (Month) (11-4/5-10/NA)	361/257/1
Leukocytes (/mm^3^)	4700 (1800–16,100)
Hemoglobin (g/dL)	13.7 (7.5–17.7)
Platelets (×10^3^/mm^3^)	151 (31–407)
AST (U/L)	48 (13–398)
ALT (U/L)	48 (10–362)
gamma-GT (U/L)	43 (8–1031)
Alpha-fetoprotein (ng/mL)	5.7 (3.6–754.7)
Total bilirubin (mg/dL)	0.7 (0.2–2.8)
Serum albumin (g/dL)	4.1 (2.5–5.1)
Total-cholesterol (mg/dL)	166 (55–310)
LDL-cholesterol (mg/dL)	94 (21–204)
HDL-cholesterol (mg/dL)	51 (8–149)
Serum creatinine (mg/dL)	0.70 (0.32–11.87)
HCV RNA (logIU/mL)	6.4 (1.2–7.8)
Fasting plasma glucose (mg/dL)	102 (65–342)
HOMA-R	2.0 (0.3–84.7)
25-hydroxyvitamin D_3_ (ng/ml)	21 (6–61)
Prothrombin time (%)	90.7 (14.5–177.6)
ISDR (0,1/others/NA)	349/158/112
Core amino acid 70 (wild type/mutant type/NA)	291/217/111
IL28B genotype rs8099917 (TT/non-TT/NA)	356/242/21
Hepatocellular carcinoma (presence/absence)	44/575

Categolic values are given as number. Continuous variable are given as median (range) *BMI* body mass index, *AST* aspartate aminotransferase, *ALT* alanine aminotransferase, *gamma-GT* gamma-glutamyltaransferase, *LDL* low-density lipoprotein cholesterol, *HDL* high-density lipoprotein cholesterol, *HOMA-R* homeostasis model assessment ratio, *ISDR* interferon sensitivity determining region, *IL28B* interleukin 28B

### Relationship between serum 25-hydroxyvitamin D_3_ levels and other factors

**Fig. 2:**
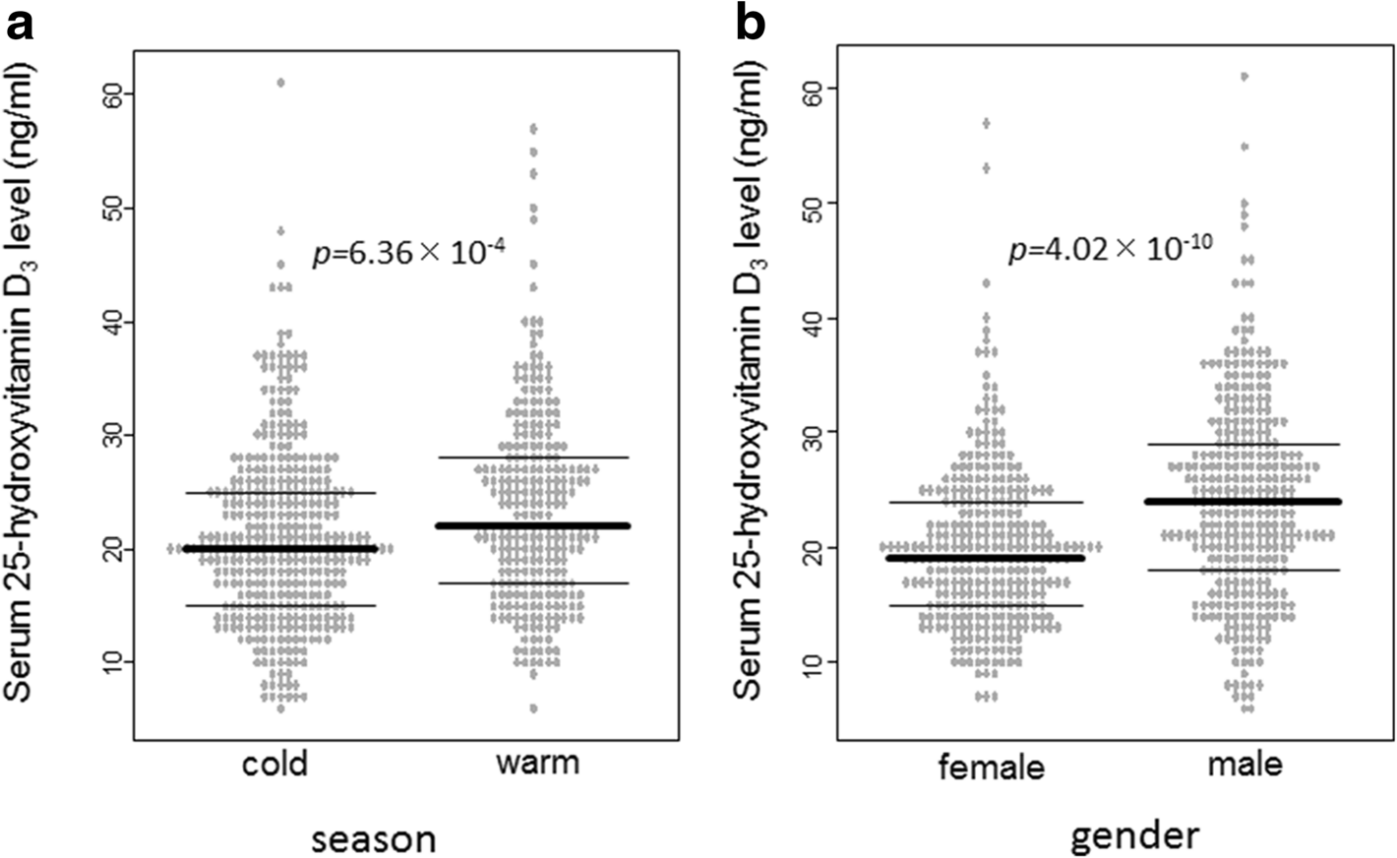
**a**) We defined the cases that serum 25-hydroxyvitamin D_3_ levels were measured between May and October as warm season, and the cases that serum 25-hydroxyvitamin D_3_ levels were measured between November and April as cold season. **b**) Serum 25-hydroxyvitamin D_3_ level according to season and gender

With regard to the season, a significant difference in serum 25-hydroxyvitamin D_3_ was observed between May–October and November–April: when the median level was 22 ng/ml [interquartile range (IQR), 17–28 ng/mL] and 20 ng/mL (IQR, 15–25 ng/mL), respectively (*p* = 6.36 × 10^−4^; Fig. 2a). As for gender, serum 25-hydroxyvitamin D_3_ levels in male patients (median, 24 ng/mL; IQR, 18–29 ng/mL) were significantly higher than those in female patients (median, 19 ng/mL; IQR, 15–24 ng/mL) (*p* = 4.02 × 10^−10^; Fig. 2b).

**Fig. 3:**
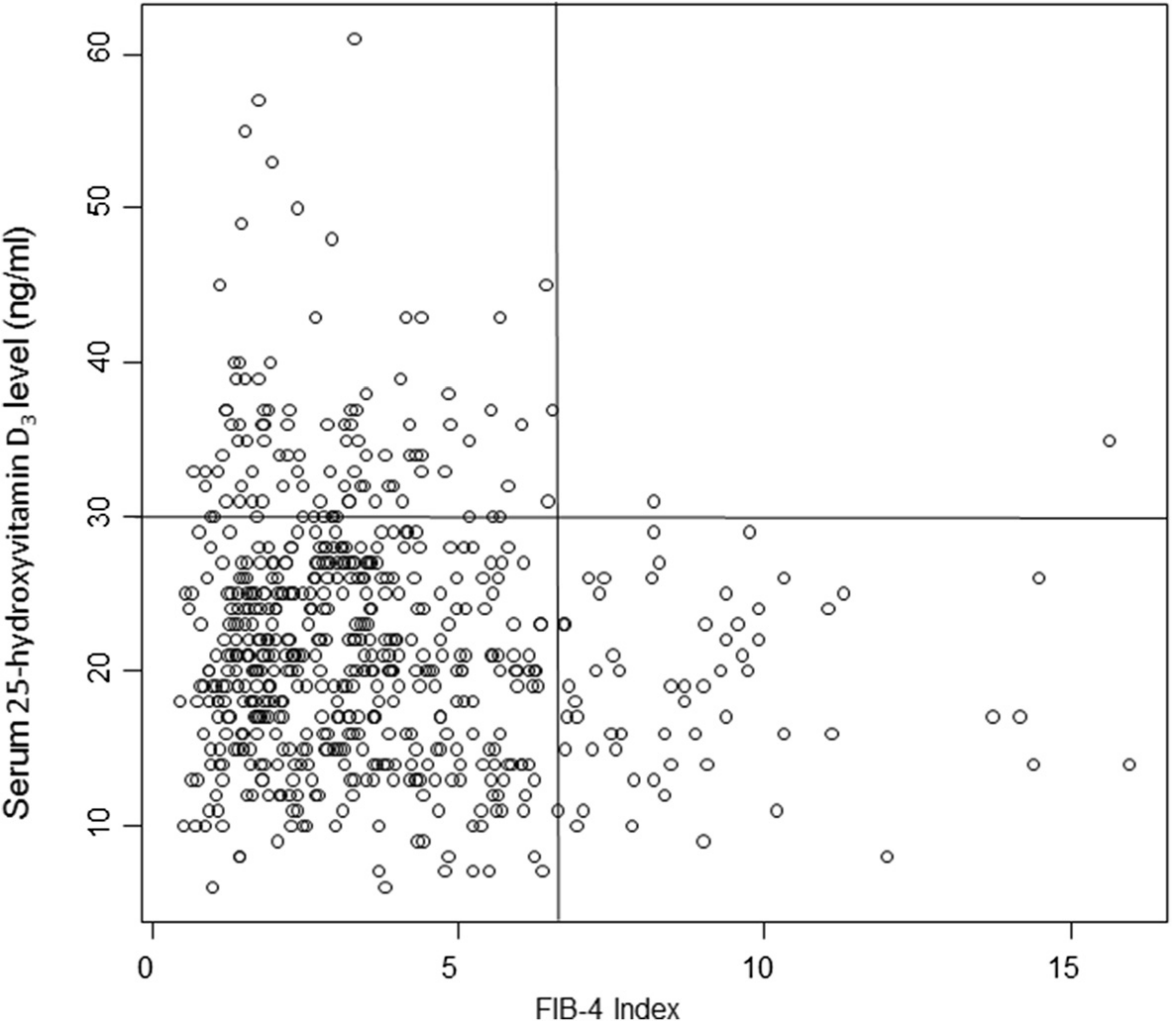
Relationship between serum 25-hydroxyvitamin D_3_ level and FIB-4 Index value. There were few patients with Fib-4 index of 7.0 or more and serum 25-hydroxyvitamin D_3_ level of 30 ng/mL or more. Conversely, 459 of 609 patients showed Fib-4 index of less than 7.0 and serum 25-hydroxyvitamin D_3_ level of less than 30 ng/mL

In the present study, the Fib-4 index was used to evaluate the degree of hepatic fibrosis. The serum 25-hydroxyvitamin D_3_ level inversely decreased with an increase in the Fib-4 index, though the negative correlation between them was weak (Fig. 3). Interestingly, there were few patients with a Fib-4 index of 7.0 or more and serum 25(OH) D_3_ level of 30 ng/mL or more (vitamin D sufficiency). Conversely, 459 of 609 (75.4 %) patients showed a Fib-4 index of less than 7.0 and serum 25-hydroxyvitamin D_3_ level of less than 30 ng/mL. When patients were categorized into two groups based on the Fib-4 index (of 3.25, which is predictive of advanced liver fibrosis), the median serum 25-hydroxyvitamin D_3_ level was 20 ng/ml (IQR, 16–26 ng/mL) in those with a Fib-4 index of greater than 3.25 and 21 ng/ml (IQR, 17–27 ng/mL) in those with a Fib-4 index of 3.25 or less (*p* = 7.52 × 10^−3^).

In univariate analysis, vitamin D deficiency was significantly influenced by the following factors: female gender [*p* = 5.49 × 10^−7^, odds ratio (OR) = 2.316, 95 % confidence interval (CI) = 1.667–3.217], high Fib-4 index (*p* = 0.031, OR = 1.076, 95 % CI = 1.007–1.150), cold season (*p* = 0.013, OR: 1.520, 95 % CI: 1.092–2.114), low hemoglobin value (*p* = 3.64 × 10^−5^, OR: 1.249, 95 % CI: 1.123–1.387), low total cholesterol (*p* = 0.012, OR: 1.006, 95 % CI: 1.001–1.012), low serum albumin (*p* = 0.001, OR: 1.898, 95 % CI: 1.278–2.817) and low prothrombin time (*p* = 0.042, OR: 1.011, 95 % CI: 1.000–1.022). In multivariate analysis, female gender (*p* = 2.03 × 10^−4^, OR = 2.290, 95 % CI = 1.479–3.545), older age (*p* = 4.30 × 10^−4^, OR = 1.038, 95 % CI = 1.017–1.060), cold season (*p* = 0.015, OR = 1.586, 95 % CI = 1.095–2.297), and low hemoglobin level (*p* = 0.037, OR = 1.165, 95 % CI = 1.009–1.345) were significantly independent factors (Table 2).

**Table 2:** Univariate and multivariate logistic regression analyses of factors associated with serum 25-hydroxyvitamin D_3_ levels deficiency in the 619 patients

		Univariate			Multivariate		
Factors	Category	OR	95 % CI	p value	OR	95 % CI	p value
Gender	Female	2.316	1.667–3.217	5.49 × 10^−7^	2.290	1.479–3.545	2.03 × 10^−4^
Age	By 1 year up	1.014	0.999–1.030	0.073	1.038	1.017–1.060	4.30 × 10^−4^
Fib-4 Index	By 0.1 up	1.076	1.007–1.150	0.031			
Season	Cold season	1.520	1.092–2.114	0.013	1.586	1.095–2.297	0.015
Hemoglobin	By 1 g/dL down	1.249	1.123–1.387	3.64 × 10^−5^	1.165	1.009–1.345	0.037
Total-cholesterol	By 1 mg/dL down	1.006	1.001–1.012	0.012			
Albumin	By 0.1 down	1.898	1.278–2.817	0.001			
Prothrombin time	By 1 % down	1.011	1.000–1.022	0.042			

Serum 25-hydroxyvitamin D_3_ levels of 20 ng/ml or less were defined as vitamin D deficiency. Multivariate analysis was performed with factors associated with serum 25-hydroxyvitamin D_3_ levels deficiency by univariate analysis (*p* < 0.10). We defined the cases that serum 25-hydroxyvitamin D_3_ levels were measured between November and April as cold season

Univariate analysis of factors contributing to serum 25-hydroxyvitamin D_3_ sufficiency identified the factors of male gender (*p* = 2.43 × 10^−7^, OR = 3.576, 95 % CI = 2.205–5.802), high body weight (*p* = 0.004, OR = 1.024, 95 % CI = 1.008–1.040), high Fib-4 index (*p* = 0.016, OR = 0.877, 95 % CI = 0.788–0.976), warm season (*p* = 0.016, OR = 1.694, 95 % CI = 1.104–2.600), high gamma-glutamyl transpeptidase (*p* = 0.029, OR = 1.002, 95 % CI = 1.000–1.005) and high serum albumin (*p* = 0.004, OR = 2.373, 95 % CI = 1.295–3.989). Multivariate analysis identified male gender (*p* = 0.001, OR = 3.400, 95 % CI = 1.635–7.069), warm season (*p* = 0.014, OR = 1.765, 95 % CI = 1.117–2.789) and serum albumin (*p* = 0.016, OR = 2.247, 95 % CI = 1.163–4.342) as independent factors (Table 3). Neither substitution of core amino acid 70 and 91 nor *IL28B* genotype, which has a strong impact on the outcome of IFN-based antiviral therapy, influenced the serum 25-hydroxyvitamin D_3_ level.

**Table 3:** Univariate and multivariate logistic regression analyses of factors associated with ≥30 ng/ml of serum 25-hydroxyvitamin D_3_ levels in the 619 patients

		Univariate			Multivariate		
Factors	Category	OR	95 % CI	p value	OR	95 % CI	p value
Gender	Male	3.576	2.205–5.802	2.43 × 10^−7^	3.400	1.949–5.918	1.60 × 10^−5^
Body weight (kg)	By 1 kg up	1.024	1.008–1.040	0.004			
Fib-4 Index	By 0.1 down	0.877	0.788–0.976	0.016			
Season	Warm season	1.694	1.104–2.600	0.016	1.765	1.117–2.789	0.015
gamma-GT (U/L)	By 1 U/ml up	1.002	1.000–1.005	0.029			
Albumin (mg/dL)	By 0.1 up	2.273	1.295–3.989	0.004	2.247	1.163–4.342	0.016
HOMA-R	By 0.1 up	0.819	0.668–1.003	0.054			

Multivariate analysis was performed with factors significantly associated with ≥30 ng/ml of serum 25-hydroxyvitamin D_3_ levels by univariate analysis (*p* < 0.10). We defined the cases that serum 25-hydroxyvitamin D_3_ levels were measured between May and October as warm season. *gamma-GT* gamma-glutamyltaransferase

## Discussion

In recent reports from various countries, the level of serum vitamin D is closely involved in the pathology of CHC and the response to IFN-based treatment of the infection. In Spain, the mean serum 25-hydroxyvitamin D_3_ level was 30.2 ng/mL in 32 CHC patients [29]. In Turkey, it was 14.16 ng/mL in 105 patients with chronic hepatitis B or C and even lower in postmenopausal female patients (11.32 ng/mL) [30]. In Italy, 73 % of 197 CHC patients showed insufficient levels of serum 25-hydroxyvitamin D_3._ In the Italian cohort, female gender was an independent factor associated with vitamin D deficiency [20]. Among 503 healthy subjects, the lowest serum 25-hydroxyvitamin D_3_ level was noted in Asians [31]. The present study was the first to investigate serum vitamin D levels and the influencing factors in a large-scale cohort of more than 600 genotype 1b CHC patients in Japan, which is located in Far East and has four distinct seasons.

Vitamin D is closely involved in host immunity. Vitamin D enhances the antigen-presenting ability of dendritic cells and increases the phagocytic capacity against external antigens [32]. Abnormal differentiation of macrophages, on which vitamin D receptors are expressed as part of the innate immune system, is observed in vitamin D-deficient mice [33]. In the acquired immune system, vitamin D receptors are expressed in mature T-cells and B-cells [34], and T-cell receptor signals are regulated by 1,25-dihydroxyvitamin D_3_ via vitamin D receptors [35]. Vitamin D also elevates the cytotoxic activity of natural killer cells [36] and has an inflammation-inhibiting action [37–39]. The incidence of tuberculosis and other infectious diseases was also reported to increase with decreased serum vitamin D levels [40].

As mentioned above, vitamin D may promote the elimination of HCV by its immunopotentiating effect. However, several studies recently reported the direct anti-HCV effect of vitamin D. In an *in vitro* study using the Huh7.5 human hepatocyte cell line, the addition of vitamin D_3_ and its metabolite 1,25-dihydroxyvitamin D_3_ decreased HCV secretion into the cell culture medium [41]. Furthermore, the concomitant administration of IFN synergistically enhanced the effect of 1,25-dihydroxyvitamin D_3_ [41]. In another *in vitro* study, 25-hydroxyvitamin D_3_ had direct anti-HCV effects by suppressing the formation of infectious HCV particles [42]. In a clinical trial involving 42 chronic hepatitis C patients, combination of 1(OH) vitamin D_3_ with pegylated IFN/ribavirin therapy augmented an early decrease in the HCV load. This effect may be ascribed to a decrease in the serum-inducible protein-10 level and enhancement of the type 1 T helper cell response [43]. Among 468 CHC patients, the decreased 25-hydroxyvitamin D level and CYP27B1-1260 promoter SNP were associated with the decreased efficacy of IFN-α-based therapy [44]. Among 42 post-liver transplant CHC patients, vitamin D supplementation was suggested to improve the antiviral treatment outcome [45]. Among 15 elderly CHC patients who received IFN-based therapy, administration of alfacalcidol, an active form of vitamin D, increased the SVR rate, particularly in female patients [46]. Among 134 HCV genotype 1 cirrhotic patients, the serum 25-hydroxyvitamin D_3_ level was an independent factor contributing to SVR with pegylated IFN/ribavirin therapy [23]. In addition, the serum 25-hydroxyvitamin D_3_ level influenced the SVR rate even in pegylated IFN/ribavirin therapy combined with telaprevir, a first-generation protease inhibitor. The impact was particularly notable in patients with unfavorable *IL28B* non-TT (rs8099917) genotype [24].

The absence of a vitamin D effect on antiviral treatment for CHC has been reported by some studies. One reported that the SVR rate did not differ according to the serum vitamin D level, whereas the complete early viral response rate was associated with it [47]. Another study reported that vitamin D supplementation for CHC patients with genotype 1b significantly increased the viral response rate at 24 weeks of treatment but did not significantly (marginally) raise the SVR rate [48]. Taken together, the pros and cons of vitamin D suggest that vitamin D supplementation in IFN-based antiviral therapy for CHC might modify the virologic response during the early treatment phase and could have an impact on treatment outcome in some groups of the patient population.

Some factors appear to have an impact on serum vitamin D levels. In this study, serum vitamin D levels were correlated inversely with serum ALT levels, suggesting the anti-inflammatory action of vitamin D [27, 37–39]. In fact, a previous study suggested that alfacalcidol supplementation could decrease serum ALT levels in CHC [46]. The progression of liver fibrosis is negatively related with the serum vitamin D level [20, 22, 44]. Our previous study showed that the median 25-hydroxyvitamin D_3_ level in 134 cirrhotic patients who received pegylated IFN/ribavirin therapy was 21 ng/mL, which was lower than that in healthy individuals and non-cirrhotic hepatitis C patients [23]. Interestingly, 1,25-dihydroxyvitamin D_3_, an active form of vitamin D, was reported to have anti-proliferative and anti-fibrotic effects on hepatic fibrosis [49]. In this study, patients with high Fib-4 index values had low serum vitamin D levels, bearing out a previously reported correlation between vitamin D and fibrosis. Of note is that this study found that almost all patients with a Fib-4 index of 7.0 or more showed serum vitamin D levels of less than 30 ng/mL (vitamin D insufficiency or deficiency). Conversely, the degree of liver fibrosis could be estimated from the serum level of vitamin D: patients with sufficient vitamin D levels would express a Fib-4 index of less than 7.0.

As described in this study, serum vitamin D levels are lower in elderly and female patients. The vitamin D deficiency in females may arise from unbalanced diets and nutrition and excess avoidance of ultraviolet light based recent esthetic trends. The high prevalence of vitamin D deficiency in Muslim women in the Middle-East [50] may be caused by their habit of extensively covering their skin with clothing, which could prevent the production of vitamin D. Serum 25-hydroxyvitamin D_3_ levels also vary depending on the season. One study from Italy showed that serum 25-hydroxyvitamin D_3_levels significantly differed between spring and winter in 211 chronic hepatitis patients, and that the season was an independent factor associated with the serum 25-hydroxyvitamin D_3_ level. Also in Japan, which has four distinct seasons, this study re-confirmed that serum 25-hydroxyvitamin D_3_ levels are lower in samples collected during the cold season. However, monthly serum levels observed in this study suggested that it might be difficult to simply divide the four seasons into two groups (warm *vs*. cold).

Decreased activity or not going out frequently may contribute to lower serum vitamin D levels. Specifically, in elderly female subjects who have anemia and/or advanced liver fibrosis, reduced sunshine exposure may result in poor vitamin D synthesis. In deed, it was shown that seasonal variation in the amount of sunlight exposure influences the serum vitamin D level: a season with low-level sunshine was an independent factor associated with a decreased serum vitamin D level, whereas, a season with more sunshine was the second most important factor associated with a sufficient serum vitamin D after sex differences. It was reported that in patients with advanced liver fibrosis, there was a decreased level of serum vitamin D. In such patients, active exposure to sunshine may increase the serum vitamin D level. Thus, our study suggests that the progression of liver fibrosis may not influence the serum vitamin D level, depending on a patient’s environmental factors. We showed another important reason which an environmental factor gives to serum vitamin D level. In this study multivariate analysis of a factor contributing to serum 25-hydroxyvitamin D_3_ sufficiency identified the factors of *warm season* as an independent factor.

There were some limitations in this study. First, the subjects were limited to HCV genotype 1 patients. In the participating facilities, most of the patients infected with minor genotype 2 have already been cured with IFN-based treatment. Therefore, this study could not clarify the influence of HCV genotype on the serum 25-hydroxyvitamin D_3_ level. Second, patients with decompensated cirrhosis were not included in the study group. They show even lower levels compared to patients with compensated cirrhosis. In addition, although we gathered seasonal data on serum vitamin D measurement, data on the number of hours of sunshine per day were not available.

## Conclusions

In summary, the present study found that serum 25-hydroxyvitamin D_3_ levels were deficient in the elderly, females, and patients with low hemoglobin level. Serum 25-hydroxyvitamin D_3_ levels were sufficient in males and high serum albumin levels. In Japan located in East Asia, serum 25-hydroxyvitamin D_3_ levels differed with the season.
